# Rare Becomes Rarer: Neuroendocrine Carcinoma of the Cervix With Synchronous Breast, Pancreatic, and Ovarian Metastases

**DOI:** 10.7759/cureus.75144

**Published:** 2024-12-05

**Authors:** Luís Guilherme Santos, Ricardo Roque, Rita Antunes Santos, Tatiana Pereira, Isabel Pazos

**Affiliations:** 1 Medical Oncology Department, Instituto Português de Oncologia de Coimbra Francisco Gentil, Coimbra, PRT

**Keywords:** breast metastasis, neuroendocrine carcinoma of cervix, ovarian metastasis, pancreatic metastasis, rare cancers

## Abstract

Neuroendocrine carcinoma of the cervix (NECC) is a rare and extremely aggressive disease. Treatment options are scarce (mainly consisting of platinum-based chemotherapy combinations), and randomized controlled trials are lacking, leading to a very poor prognosis. It is prone to early metastasis, often with more than one affected site at diagnosis. The lung, liver, brain, and bone are the most frequent sites. We present the case of a 33-year-old female patient with no significant medical history. Postcoital bleeding led to the diagnosis of human papillomavirus (HPV)-related NECC after a tissue biopsy of a cervical mass. Disease staging showed suspected metastasis on the breast, pancreas, and ovaries, each of which is very rarely described in the literature. Both the breast and ovarian metastases were histologically confirmed through biopsy, while pancreatic metastases were deemed highly likely based on radiological findings. The patient was started on first-line chemotherapy with significant toxicities and rapid disease progression and is currently on third-line chemotherapy treatment. To our knowledge, this is the first reported case of these very rare sites of metastasis from NECC being present simultaneously, highlighting the complexity of this challenging entity.

## Introduction

Neuroendocrine carcinoma of the cervix (NECC) is a rare, poorly studied, and extremely aggressive form of cervical cancer. It accounts for approximately 1.4% of all cervical cancers and has been often linked to human papillomavirus (HPV) infection [1]. NECC is associated with multiple early metastases, with the liver, lungs, brain, and bone being frequently described as the most common sites [2]. Metastases to other sites are very rare. Prospective randomized controlled trials addressing its treatment are difficult due to the disease's very low incidence [3], and prognosis remains extremely poor, with median five-year survival rates of 0%-15% for advanced-stage disease [1].

## Case presentation

A 33-year-old female patient with no relevant prior medical history, Eastern Cooperative Oncology Group Performance Scale (ECOG-PS) 0, was first observed in the Emergency Department due to postcoital bleeding and suprapubic discomfort for the previous four months. Gynecological examination showed an exophytic, 5x5 cm lesion arising from the cervix, apparently invading the parametrium. Physical examination was otherwise normal. Bloodwork showed no relevant alterations (complete blood count and liver and renal function tests were all under normal limits). Since it was readily available at that time, a thoraco-abdominal-pelvic computed tomography (CT) scan was requested for an initial workup, showing a suspected cervical lesion with no other alterations. A biopsy was performed, revealing a malignant, poorly differentiated small-cell neoplasm with diffuse positivity for neuroendocrine markers (CD56, chromogranin A, synaptophysin) and positivity for p16 and Ki67 at 100%. Cytology for HPV 16 was positive. A diagnosis of small-cell NECC was made.

**Figure 1:**
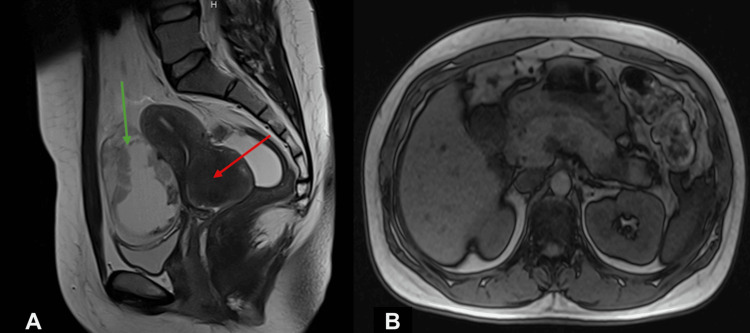
Abdominopelvic MRI (A) Abdominopelvic MRI showing a posterior-lateral cervical mass (red arrow) and concomitant, extensive, heterogenous, mainly cystic mass (green arrow) apparently arising from the left ovary; (B) abdominal MRI showing pancreatic edema with multiple nodular lesions compatible with metastasis from the extra-pancreatic site.

Magnetic resonance imaging (MRI) of the pelvis was requested to address locoregional extension. A positron-emission tomography (PET) scan was also performed to address possible metastatic disease, confirming hypermetabolic lesions on the cervix while also revealing apparent lesions on the right breast, left ovary, and pancreas. After discussion, an abdominal MRI was also added to further clarify the abdominal lesions. Pelvic and abdominal MRIs are shown in Figure 1.

**Figure 2:**
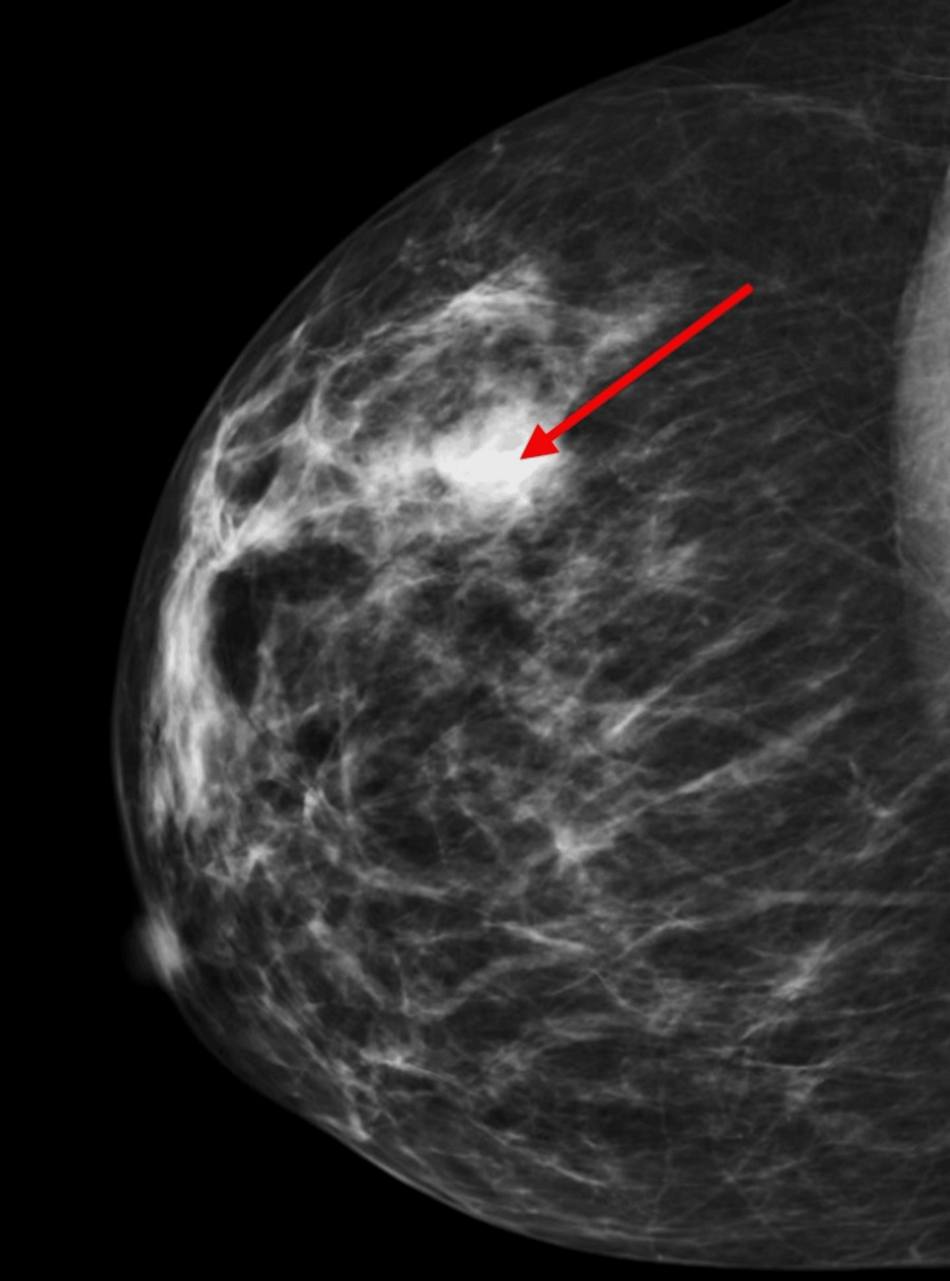
Mammogram Mammogram showing a lesion on the right breast (red arrow).

Pelvic MRI (Figure 1A) confirmed a locally advanced cervical lesion invading the uterine isthmus and additionally showed a heterogeneous mass apparently arising from the left ovary, suspicious of concomitant ovarian cystadenocarcinoma. However, a biopsy of this mass was performed, confirming metastasis of neuroendocrine origin. Abdominal MRI (Figure 1B) showed multiple, diffuse nodular lesions throughout the pancreas, which, due to their multiplicity and radiological characteristics (diffusion restriction and vascular enhancement in arterial phase), were more in favor of metastization from a neuroendocrine primary rather than a pancreatic primary. Additionally, the right breast lesion detected in PET was further studied with a mammogram (Figure 2) and subjected to biopsy, again confirming neuroendocrine origin.

After a multidisciplinary discussion, the patient was started on first-line palliative chemotherapy with etoposide 100 mg/m^2^ (days 1, 2, and 3) plus cisplatin 75 mg/m^2^ (day 1) every 21 days. Serum chromogranin A was highly elevated at this point (2009 ng/ml). The patient completed a total of four cycles with hematological toxicity, namely, grade 3 anemia requiring blood transfusions. Clinical response was poor, with significant abdominal distension and a steady increase in tumor markers (chromogranin A 8042 ng/ml).

**Figure 3:**
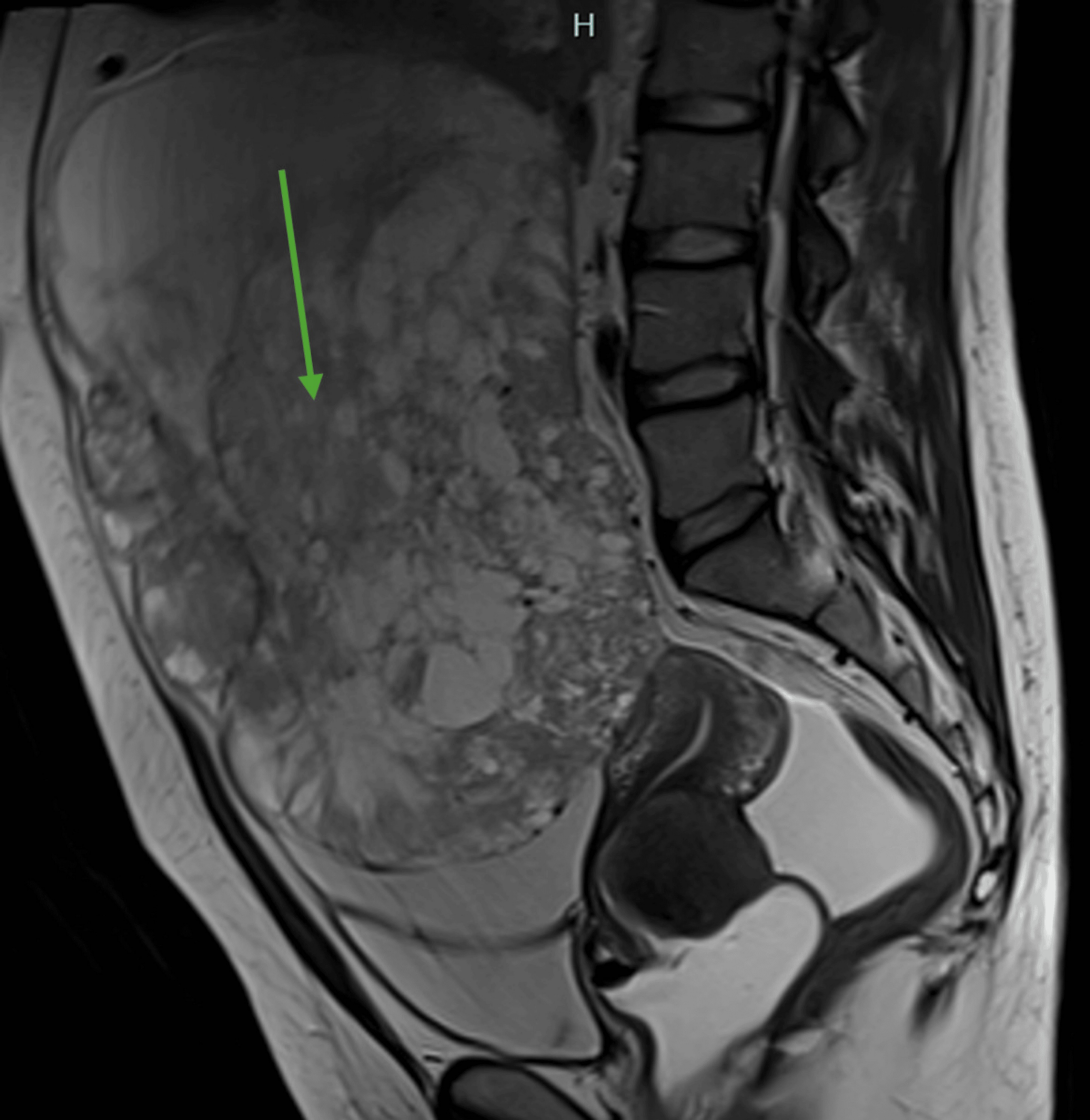
Abdominal MRI This MRI shows disease progression on first-line treatment. The mass previously visible arising from the left ovary (green arrow) now extends far beyond the pelvic cavity and into the abdomen. A cervical mass is also still present.

An MRI was repeated after approximately three months (Figure 3), which confirmed disease progression with an extensive mass now occupying the pelvic and abdominal cavity almost entirely while also showing possible bone metastasis. Topotecan 0.75 mg/m^2^ plus paclitaxel 175 mg/m^2^ plus bevacizumab 15 mg/kg every 21 days was started as a second-line therapy. The patient completed a total of three cycles with significant (grade 3) treatment-related skin toxicity requiring hospital admission. The radiological evaluation of response after three cycles again showed disease progression (increased volume of the abdominal masses and ascites), compatible with clinical signs and symptoms (uncontrolled pain and worsened abdominal distension). However, the patient maintained an ECOG-PS of 1 and was thus started on third-line FOLFOX (oxaliplatin 85 mg/m^2^ on day 1 plus leucovorin 400 mg/m^2^ on day 1 plus 5-fluorouracil 400 mg/m^2^ on day 1 followed by 1200 mg/m^2^ continuous infusion on days 1 and 2, every 14 days), completing three cycles until the present day.

## Discussion

Accounting for approximately 1.4% of all cervical cancers, NECC is a rare disease mainly described in literature through case reports and case series. Its pathogenesis has been linked to the oncogenic nature of HPV, mainly HPV serotype 18. HPV infection was present in our patient, although in our case, the associated strain was HPV 16. Diagnosis is through biopsy and immunohistochemistry-based detection of neuroendocrine markers. Poorly differentiated small-cell NECC is the most common histological variety [1].

NECC is an extremely aggressive disease, being associated with early lympho-vascular spread and early metastasis, with most cases being diagnosed at advanced stages and frequently with multiple sites of metastasis. A population-based study [2] found that 44.4% of patients with NECC had more than one metastatic site at diagnosis, with the lung, liver, brain, and bone being the most frequent.

Breast metastasis from extramammary primaries is considered rare, with reported incidences as low as 0.4% [4]. Breast metastasis arising from cervical cancers is even rarer: a case study from 2022 [4] was, to our knowledge, the second-ever reported case. Pancreatic metastasis from cervical cancer is extremely rare as well, with recent database searches reporting as few as four cases [5]. The main challenge associated with pancreatic metastasis seems to be distinguishing them from pancreatic primaries, which are far more common; in our case, radiological characteristics strongly supported metastasis instead of a concomitant primary. As for ovarian metastasis, a study that included 86 patients with NECC [6] found incidence rates of 3.5%, and as such, they are also considered very atypical. Interestingly, aside from suspicion of two small bone metastases, none of the other most common sites were present in this patient.

Treatment for NECC is challenging due to its low incidence and lack of prospective randomized controlled trials. First-line treatment is often extrapolated from treatment of other neuroendocrine sites and includes platinum-based chemotherapy duplets. Later lines of treatment are less defined, and no standard of care exists. These challenges explain the extremely grim prognosis of this entity, especially considering advanced-stage disease.

## Conclusions

NECC is a rare and extremely aggressive disease associated with a poor prognosis and limited treatment options, making inclusion in clinical trials of the utmost importance. To our knowledge, this is the first-ever reported case of simultaneous breast, pancreatic, and ovarian metastases from NECC, with each specific site being extremely rare individually. Our case highlights the complexity of diagnosing and managing this rare cancer and the need for further studies on the subject.

## References

[1] Tangella AV, Yadlapalli DC (2023). Neuroendocrine carcinoma of cervix: a case series. Cureus.

[2] Li Q, Yu J, Yi H, Lan Q (2022). Distant organ metastasis patterns and prognosis of neuroendocrine cervical carcinoma: a population-based retrospective study. Front Endocrinol (Lausanne).

[3] Verma S, Dubey H, Gupta S, Ranjan A, Goel H, Sharma A (2023). Neuroendocrine carcinoma of cervix and review literature. Int J Surg Case Rep.

[4] Sapiai NA, Abdul Muthalib F, Mat Idris NA, Mohd Nasir ZA (2022). A rare case of metastatic small cell carcinoma of breast from mixed types of cervical carcinoma. Radiol Case Rep.

[5] Wang Y, Hong W, Lu J (2024). Pancreatic metastasis from small-cell neuroendocrine cervical cancer - a case report and literature review. Ann Clin Case Rep.

[6] Xiang X, Zhang Y, Hua K, Ding J (2023). Impacts of ovarian preservation on the prognosis of neuroendocrine cervical carcinoma: a retrospective analysis based on machine learning. World J Surg Oncol.

